# Performance of Shear Wave Elastography for Differentiation of Benign and Malignant Solid Breast Masses

**DOI:** 10.1371/journal.pone.0076322

**Published:** 2013-10-18

**Authors:** Guiling Li, De-Wei Li, Yu-Xiao Fang, Yi-Jiang Song, Zhu-Jun Deng, Jian Gao, Yan Xie, Tian-Sheng Yin, Li Ying, Kai-Fu Tang

**Affiliations:** 1 Institute of Genomic Medicine, Wenzhou Medical University, Wenzhou, P.R. China; 2 Department of Hepatobiliary Surgery, First Affiliated Hospital, Chongqing Medical University, Chongqing, P.R. China; 3 Department of Gastroenterology, Second Affiliated Hospital, Chongqing, P.R. China; 4 Department of Ultrasonography, First Affiliated Hospital of Wenzhou Medical University, Wenzhou 325000, P.R. China; University of North Carolina School of Medicine, United States of America

## Abstract

**Objectives:**

To perform a meta-analysis assessing the ability of shear wave elastography (SWE) to identify malignant breast masses.

**Methods:**

PubMed, the Cochrane Library, and the ISI Web of Knowledge were searched for studies evaluating the accuracy of SWE for identifying malignant breast masses. The diagnostic accuracy of SWE was evaluated according to sensitivity, specificity, and hierarchical summary receiver operating characteristic (HSROC) curves. An analysis was also performed according to the SWE mode used: supersonic shear imaging (SSI) and the acoustic radiation force impulse (ARFI) technique. The clinical utility of SWE for identifying malignant breast masses was evaluated using analysis of Fagan plot.

**Results:**

A total of 9 studies, including 1888 women and 2000 breast masses, were analyzed. Summary sensitivities and specificities were 0.91 (95% confidence interval [CI], 0.88–0.94) and 0.82 (95% CI, 0.75–0.87) by SSI and 0.89 (95% CI, 0.81–0.94) and 0.91 (95% CI, 0.84–0.95) by ARFI, respectively. The HSROCs for SSI and ARFI were 0.92 (95% CI, 0.90–0.94) and 0.96 (95% CI, 0.93–0.97), respectively. SSI and ARFI were both very informative, with probabilities of 83% and 91%, respectively, for correctly differentiating between benign and malignant breast masses following a “positive” measurement (over the threshold value) and probabilities of disease as low as 10% and 11%, respectively, following a “negative” measurement (below the threshold value) when the pre-test probability was 50%.

**Conclusions:**

SWE could be used as a good identification tool for the classification of breast masses.

## Introduction

Over the last 10 years, elastography has been used in addition to B-mode ultrasonography to identify malignant breast masses [1], [2], [3], [4], [5]. Elasticity measurements have been reported to be useful for the diagnosis of malignant breast masses, which are usually stiffer than benign or normal soft tissues [6], [7], [8]. Elasticity imaging can increase B-mode accuracy and specificity in differentiating between benign and malignant masses, as well as in reducing the numbers of biopsies performed in patients with benign masses [9].

Improvements in elasticity techniques can result in improved characterization of tissue, thus improving patient diagnosis [10]. Shear wave elastography (SWE) uses the acoustic radiation force induced by ultrasound beams to perturb underlying tissues, with the propagation of the resulting shear waves recorded in real time by ultrafast imaging [11]. Determination of local shear wave velocity yields a two-dimensional map of shear elasticity. Thus, in contrast to free-hand ultrasound elastography, with the application of manual compression, SWE is operator-independent, reproducible, and quantitative [11]. Moreover, SWE uses a conventional linear array probe, allowing its incorporation into standard diagnostic ultrasonographic examinations. Two different SWE modes are currently available, supersonic shear imaging (SSI) [12] and the acoustic radiation force impulse (ARFI) technique [13]. Both modes induce mechanical vibrations by using an acoustic radiation force created by a focused ultrasound beam. The propagation of shear waves can be captured by a very fast ultrasound acquisition sequence. Unlike the ARFI technique, stiffness information from SSI is reported in kPa.

Although several studies have reported the diagnostic value of SWE for differentiating between benign and malignant breast masses, those studies reported wide ranges of sensitivity (75.6–97%) [12], [14], [15] and specificity (75.1–95.1%) [11], [13], [16], [17]. To clarify the diagnostic accuracy of this technique, we performed a meta-analysis assessing the performance of SWE in the classification of breast masses.

## Materials and Methods

### Search strategy

PubMed, EMBASE, the Cochrane Library, and the ISI Web of Knowledge were searched for studies, published in English before November 30, 2012, using the following search terms: (elastography OR sonoelastography OR elasticity imaging OR shear wave elastography OR supersonic shear imaging OR acoustic radiation force impulse OR ARFI) AND (breast OR breast masses OR breast neoplasms) AND (diagnosis OR diagnostic test). In order to retrieve more related articles, article type was not included in the search strategy. We also manually searched references in key articles. The study was performed in accordance with the PRISMA statement [18].

### Selection criteria

To be included, studies had to meet the following 3 criteria. First, each had to evaluate the performance of SWE in differentiating between benign and malignant breast masses using cytological examinations of fine needle aspiration biopsy samples or histological examination of surgically removed tissue (the diagnostic reference standard). Second, each study had to report the data needed to calculate the true positive, false positive, true negative, and false negative rates of SWE for the differentiation between benign and malignant breast masses. If such data were unavailable, the corresponding author was contacted via e-mail and invited to provide them; if the author failed to reply, the study was excluded. Third, each study had to include at least 30 patients, because smaller studies are less reliable. If 2 or more studies evaluated overlapping patient samples, only the study with the larger number of patients was included.

### Study selection and data extraction

The eligible studies were assessed and reciprocally verified independently by 2 reviewers who were long engaged in research on breast cancer diagnosis with similar experience in this kind of activity; disagreements were resolved in consultation with a third investigator. Using a fixed protocol, the 2 investigators independently extracted data from each study, including the lead author, publication year, region, patient ages, numbers of patients, numbers of breast masses, percentages of malignant masses, mean tumor diameters, diagnostic standards, cut-off values of the methods, SWE mode, and study quality. True positive, false positive, true negative, and false negative were also extracted, allowing the sensitivity, specificity, positive predictive value (PPV), and negative predictive value (NPV) of each reported test threshold to be calculated.

### Quality assessment

The quality assessment of diagnostic accuracy studies (QUADAS) questionnaire was used to assess the quality of the studies included in the meta-analysis [19]. This questionnaire was designed to assess the internal and external validity of the diagnostic accuracy of studies included in systematic reviews. The QUADAS tool has 14 items that assess study design-related issues and the validity of the study results. Each item was scored “yes” if reported, “no” if not reported, or “unclear” if adequate information was not available in the article.

### Data synthesis and statistical analysis

Summary sensitivities and specificities and diagnostic odds ratios (DOR), with corresponding 95% confidence intervals (CI), were calculated for the ability of SWE to accurately diagnose malignant breast masses. The DOR, calculated as the positive likelihood ratio (PLR) divided by the negative likelihood ratio (NLR), represents the likelihood of a condition existing in a person with a positive test result, relative to a person with a negative test result. Furthermore, it is a single indicator of test performance (like accuracy) but is independent of prevalence (unlike accuracy) and is presented as an odds ratio, a measure that is familiar to medical practitioners. The PLR is a measure of the likelihood that a positive SWE result (malignant breast mass present) would occur in a patient with an actual malignant breast mass, whereas the NLR is a measure of the likelihood that a negative SWE result (absence of a malignant breast mass) would occur in a patient without a malignant breast mass. A hierarchical summary receiver operating characteristic (HSROC) curve was also plotted. Between-study heterogeneity was evaluated by computing Higgins I^2^ and χ^2^ tests for heterogeneity, using the generic inverse variance method of the meta-analysis of DOR. An I^2^ value of more than 50% or a χ^2^ P-value of 0.10 was considered substantial heterogeneity. If the primary studies were heterogeneous, the random effects method was used for the pooled analyses.

In addition, to explore sources of heterogeneity among studies, a meta-regression technique was used with the following predefined characteristics: prevalence of malignant breast masses, median patient age, mean mass size, and QUADAS score. A P value less than 0.05 was considered statistically significant. To identify any publication bias, Deeks' funnel plot asymmetry test was performed, allowing formal testing for publication bias by the regression of diagnostic log odds ratio against 1/sqrt (effective sample size), weighted by effective sample size, with P<0.10 for the slope coefficient indicating significant asymmetry [20]. An analysis was performed according to the SWE mode used, SSI or ARFI.

The clinical utility of a diagnostic test can be assessed using analysis of Fagan plot [21]. Pre-test probabilities (Ppre, suspicion for malignant breast masses) of 25%, 50%, and 75% were compared with their corresponding post-test probabilities (Ppost) of malignant breast masses following a “positive” or “negative” SWE result, based on the overall sensitivity and specificity. The post-test probability of malignant breast masses was calculated from likelihood ratios (LRs), using Bayes theorem, with Ppost = (LR×Ppre)/[(1−Ppre)×(1−LR)]. “Positive” and “negative” SWE results were defined as all results above and below the defined diagnostic standard for malignant breast masses in each individual study, respectively.

All statistical analyses were performed using the MIDAS and METANDI modules in Stata 11.0 (Stata, College Station, TX).

## Results

### Search results and study characteristics

**Figure 1:**
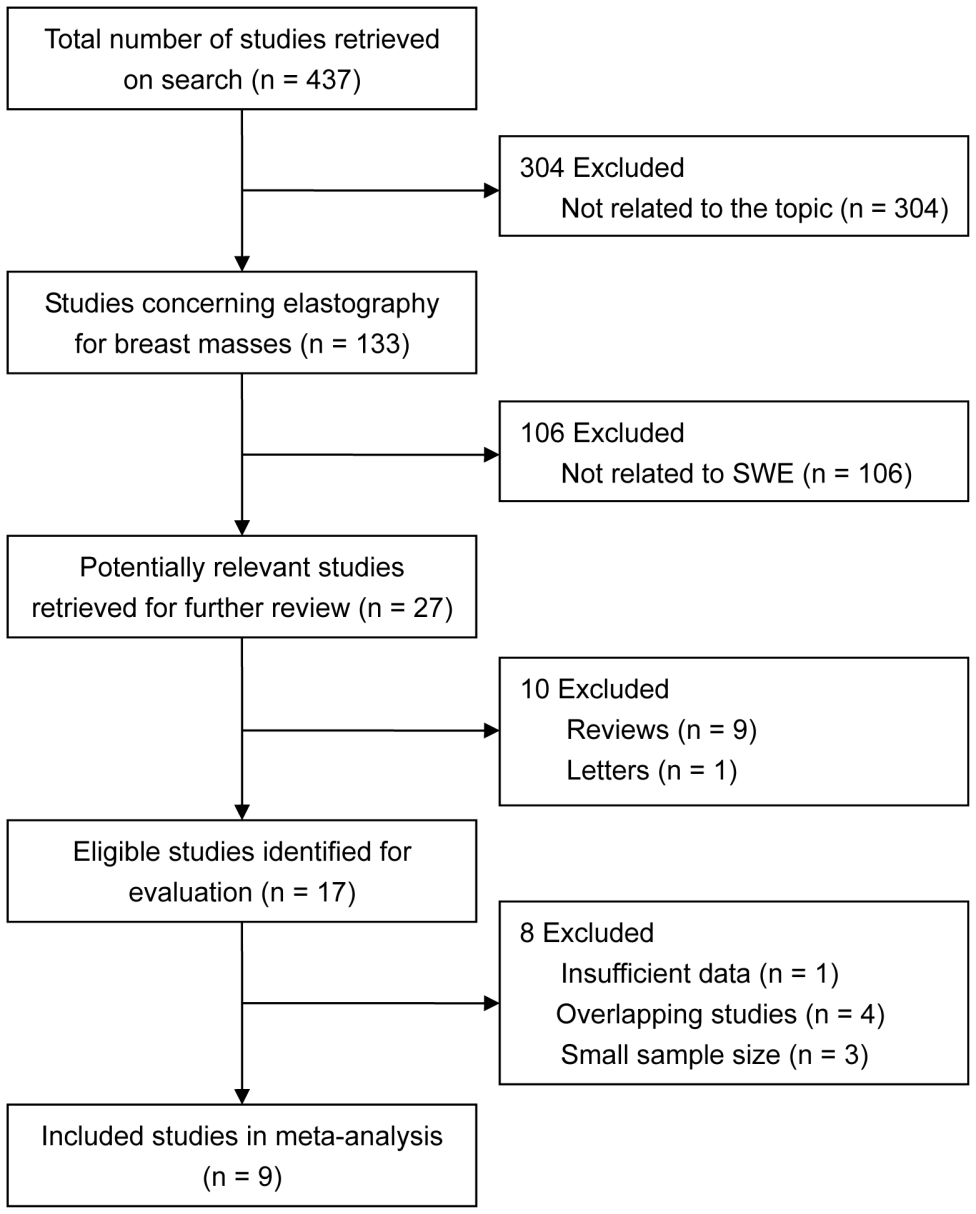
Study selection flow chart. SWE, shear wave elastography.

The described search strategies retrieved a total of 437 studies. Three hundred and four studies were eliminated because they were not related to the topic. Of the remaining 133 studies on elastography for the detection of breast masses, 106 were not related to SWE. Therefore, 27 potentially relevant studies were identified for further evaluation. In these studies, the study title and abstract may have indicated that the study evaluated the accuracy of SWE for identifying malignant breast masses; however, not all of the study inclusion criteria may have been met. Ultimately, 18 of these studies were excluded, 10 because they were undesirable article types, 1 because of insufficient data [10], and 3 because of small sample sizes [22], [23], [24]. The studies by Tozaki et al. [25], [26], [27] and Lee et al. [28] were also excluded because their patient samples overlapped with the studies performed by Tozaki et al. [4] and Gweon et al. [29], respectively. Thus, 9 studies fulfilled our inclusion criteria. Data on the entire study population of Berg et al. were received by email [12]. A flowchart describing the study selection is shown in Figure 1.

The main characteristics of studies included in the meta-analyses are summarized in Table 1. These 9 studies included 2000 breast masses (1230 benign, 770 malignant) in 1888 patients (median age, 50.2 years). The overall prevalence of malignant breast masses was 38.5% (range, 27.1–63.4%). Among the included studies, 5 used the SSI mode [11], [12], [16], [29], [30] and 4 used the ARFI mode [4], [13], [14], [17]. The QUADAS scale showed that the included studies were of good methodological quality (Table 2), with each meeting over 10 of the QUADAS requirements.

**Table 1:** Main characteristics of studies evaluating the performance of shear wave elastography in differentiating between benign and malignant breast masses.

Author, Year	Ref.	Country	No. of patients	No. of breast masses	Median age (year, range)	Malignant breast masses rate (%)	Mean mass size (cm)	Reference standard	Diagnostic standard	Cutoff	QUADAS Score
Evans, 2010	16	UK	52	53	44 (18–84)	56.6	2.00	Pathology	SSI	50 Kpa	11
Chang, 2011	11	Korea	158	182	48.1 (22–79)	48.9	1.76	Pathology	SSI	80.17 Kpa	11
Meng, 2011	17	China	86	92	45.6 (17–78)	29.3	2.57	Pathology	ARFI	6.37 m/s	10
Bai, 2012	14	China	108	143	44 (19–87)	28.7	2.01	Pathology	ARFI	3.07 m/s	11
Berg, 2012	12	USA	939	939	52 (21–94)	30.8	1.37	Pathology	SSI	80 Kpa	13
Evans, 2012	30	UK	173	175	56 (18–94)	63.4	1.92	Pathology	SSI	155 Kpa	11
Gweon, 2012	15	Korea	119	133	45.3 (21–77)	27.1	1.38	Pathology	SSI	12.1*	10
Jin, 2012	13	China	95	122	43.5 (18–69)	45.9	2.26	Pathology	ARFI	3.65 m/s	11
Tozaki, 2012	23	Japan	158	161	52 (26–80)	56.5	1.41	Pathology	ARFI	3.5 m/s	11

ARFI, acoustic radiation force impulse; QUADAS, quality assessment of diagnostic accuracy studies; SSI, supersonic shear imaging.

* Standard deviation as the threshold.

**Table 2:** Quality assessment of included studies.

	Q1	Q2	Q3	Q4	Q5	Q6	Q7	Q8	Q9	Q10	Q11	Q12	Q13	Q14
Author, Year	Spectrum composition	Selection criteria	Appropriate reference standard	Disease progression bias	Partial verification bias	Differential verification bias	Incorporation bias	Test execution details	Reference execution details	Test review bias	Diagnostic review bias	Clinical review bias	Intermediate results	Withdrawals
Evans, 2010	Yes	Yes	Yes	Unclear	Yes	Yes	Yes	Yes	Yes	Unclear	Unclear	Yes	Yes	Yes
Chang, 2011	Yes	Yes	Yes	Unclear	Yes	Yes	Yes	Yes	Yes	Unclear	Unclear	Yes	Yes	Yes
Meng, 2011	Yes	No	Yes	Unclear	Yes	Yes	Yes	Yes	Yes	Unclear	Unclear	Yes	Yes	Yes
Bai, 2012	Yes	Yes	Yes	Unclear	Yes	Yes	Yes	Yes	Yes	Unclear	Unclear	Yes	Yes	Yes
Berg, 2012	Yes	Yes	Yes	Unclear	Yes	Yes	Yes	Yes	Yes	Yes	Yes	Yes	Yes	Yes
Evans, 2012	Yes	Yes	Yes	Unclear	Yes	Yes	Yes	Yes	Yes	Unclear	Unclear	Yes	Yes	Yes
Gweon, 2012	Yes	No	Yes	Unclear	Yes	Yes	Yes	Yes	Yes	Unclear	Unclear	Yes	Yes	Yes
Jin, 2012	Yes	No	Yes	Unclear	Yes	Yes	Yes	Yes	Yes	Unclear	Unclear	Yes	Yes	Yes
Tozaki, 2012	Yes	No	Yes	Unclear	Yes	Yes	Yes	Yes	Yes	Unclear	Unclear	Yes	Yes	Yes

### Diagnostic accuracy of SSI

**Figure 2:**
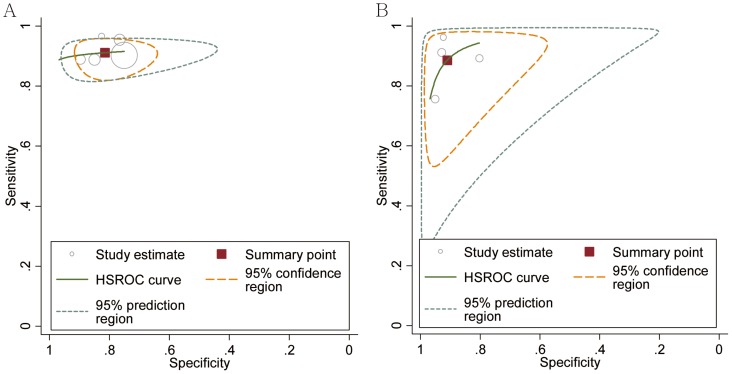
Hierarchical summary receiver operating characteristic curve of shear wave elastography in the differentiation of breast masses. The size of the dots for 1 - specificity and sensitivity of the single studies in the ROC space was derived from the respective sample size.

Five studies evaluated the diagnostic accuracy of SSI for the differentiation of benign from malignant breast masses (Table 1). The summary sensitivity and specificity were 0.91 (95% CI, 0.88–0.94) and 0.82 (95% CI, 0.75–0.87), respectively (Table 3). The HSROC was 0.92 (95% CI, 0.90–0.94) (Figure 2A). On the basis of these values and assuming 37.4% malignant breast masses (as observed in the included studies), the PPV and NPV were 0.82 (95% CI, 0.77–0.86) and 0.89 (95% CI, 0.84–0.93), respectively (Table 3). Table 3 showed the main results of SSI for the detection of malignant breast masses. The pooled accuracy was 82.9%. There was statistically significant heterogeneity in the diagnostic odds ratios (Table 3). However, according to the meta-regression analysis, the SSI accuracy for identifying malignant breast masses was not affected by any covariate. Significant publication bias existed among these studies (P = 0.001) (Figure S1A).

**Table 3:** Subgroup analysis of the accuracy of shear wave elastography modes in differentiating between benign and malignant breast masses.

SWE mode	Studies, N	Sensitivity (95%CI)	Specificity (95%CI)	DOR (95%CI)	HSROC (95%CI)	PPV (95%CI)	NPV (95%CI)	PLR (95%CI)	NLR (95%CI)	I^2^	p	Pre-test	Post test (+)	Post test (−)
SSI	5	0.91 (0.88–0.94)	0.82 (0.75–0.87)	45.71 (26.13–79.97)	0.92 (0.90–0.94)	0.82 (0.77–0.86)	0.89 (0.84–0.93)	4.95 (3.53–6.93)	0.11 (0.08–0.15)	99.9%	0.001	25%	62%	3%
												50%	83%	10%
												75%	94%	25%
ARFI	4	0.89 (0.81–0.94)	0.91 (0.84–0.95)	79.77 (39.16–162.48)	0.96 (0.93–0.97)	0.90 (0.86–0.94)	0.88 (0.83–0.92)	9.95 (5.66–17.50)	0.12 (0.07–0.21)	84.1%	0.001	25%	77%	4%
												50%	91%	11%
												75%	97%	27%

ARFI, acoustic radiation force impulse; CI, confidence interval; DOR, diagnostic odds ratio; HSROC, hierarchical summary receiver operating characteristic; NLR, negative likelihood ratio; NPV, negative predictive value; PLR, positive likelihood ratio; PPV, positive predictive value; SSI, supersonic shear imaging.

**Figure 3:**
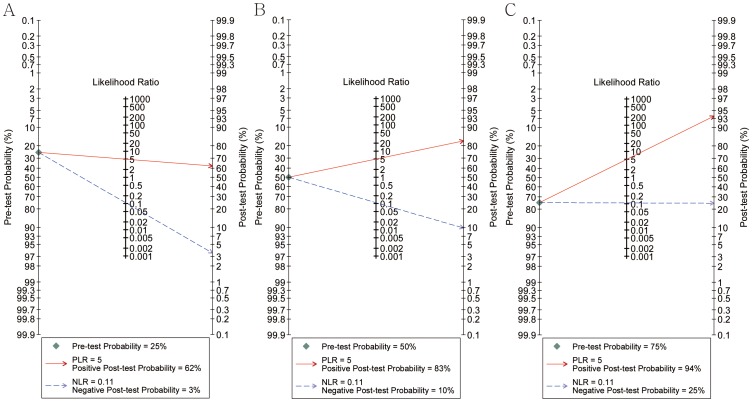
Analysis of the Fagan plot to evaluate the clinical utility of supersonic shear imaging (SSI) in differentiating benign from malignant breast masses. (A) At a pre-test probability for malignant breast masses of 25% (low clinical suspicion), the post-test probability of malignancy with a negative SSI result was 3%; this could be considered sufficient to rule out malignancy. (B) At a pre-test probability for malignant breast masses of 50% (worst-case scenario), the post-test probabilities of malignancy with positive and negative SSI results were 83% and 10%, respectively, indicating the usefulness of this test. (C) At a pre-test probability for malignant breast masses of 75% (high clinical suspicion), the post-test probability of malignancy, with a positive SSI result, was 94%; thus, a positive SSI result could be considered sufficient for a diagnosis of malignancy. A Fagan plot consists of a vertical axis on the left showing the pre-test probability, an axis in the middle representing the likelihood ratio, and a vertical axis on the right showing the post-test probability. NLR, negative likelihood ratio; PLR, positive likelihood ratio.

The analysis of the Fagan plot demonstrated that SSI was very informative, with an 83% probability of correctly detecting malignant breast masses following a “positive” measurement when the pre-test probability was 50%, and the probability of disease was as low as 10% following a “negative” measurement. However, when the pre-test probability was 25%, SSI only had a 62% probability of correctly diagnosing malignant breast masses following a “positive” measurement. In addition, the diagnosis would be wrong in 25% of patients with a “negative” measurement when the pre-test probability was 75%, although the probability of a correct diagnosis following a “positive” measurement exceeded 90% for malignant breast masses (Figure 3, Table 3).

### Diagnostic accuracy of ARFI

The diagnostic accuracy of ARFI for differentiating between benign and malignant breast masses was evaluated in 4 studies (Table 1). The summary sensitivity and specificity were 0.89 (95% CI, 0.81–0.94) and 0.91 (95% CI, 0.84–0.95), respectively (Table 3). The HSROC was 0.96 (95% CI, 0.93–0.97) (Figure 2B). Based on these values, and assuming 41.5% of the breast masses were malignant (as observed in the included studies), the PPV and NPV were 0.90 (95% CI, 0.86–0.94) and 0.88 (95% CI, 0.83–0.92), respectively (Table 3). The main results of ARFI for the detection of malignant breast masses were shown in Table 3. The pooled accuracy was 89.8%, which was not significantly higher than that for SSI (P = 0.288). There was statistically significant heterogeneity in the diagnostic odds ratios (Table 3). However, according to the meta-regression analysis, the accuracy of ARFI for detecting malignant breast masses was not affected by any covariate. No publication bias existed among these studies (P = 0.66) (Figure S1B).

ARFI was also very informative, with a probability of establishing a correct diagnosis following a “positive” measurement reaching 91% for the differentiation of benign from malignant breast masses when pre-test probability was 50%, and the probability of disease was as low as 11% following a “negative” measurement. Although ARFI was very informative, it lowered the negative post-probability of malignant breast masses to as low as 4% from a pre-probability of 25% when a “negative” measurement was obtained. However, only a 77% probability of correctly diagnosing malignant breast masses following a “positive” measurement was observed. For a pre-test probability of 75%, the probability of a correct diagnosis following a “positive” measurement reached 97% for malignant breast masses, but the diagnosis would be wrong for 27% of patients with a “negative” measurement (Figure S2, Table 3).

## Discussion

SWE differs from conventional elastography in that the radiation force of the ultrasound beams induces the mechanical vibrations of SWE automatically. The reliability of SWE does not depend on the ability of the sonologist to correctly vibrate or stress the tissue [31]. Therefore, SWE overcomes the intrinsic limitations of conventional elastography, which can only determine qualitative and relative elasticity. SWE can therefore be considered as a quantitative diagnostic tool for breast cancer [32], [33].

In this meta-analysis, we evaluated the ability of SWE to differentiate between benign and malignant breast masses. Our results indicated that SWE had a high accuracy for differentiating benign breast masses from malignant ones. The HSROCs for the diagnosis of malignant breast masses by SSI and ARFI were 0.92 (95% CI, 0.90–0.94) and 0.96 (95% CI, 0.93–0.97), respectively. Furthermore, the reproducibility of diagnostic tests across observers is another important consideration. In previous studies, the intraclass correlation coefficient for SWE analysis agreement between 2 operators was more than 0.85, based on the averaged values from 2 images acquired by each operator [16], [30], [31]. Moreover, SWE does not require compression of tissues during elasticity examination. Thus, SWE is a reliable and noninvasive procedure and can be easily and inexpensively be integrated into current imaging protocols using conventional ultrasonography. The present study suggested that SWE classification was at least as accurate as B-mode ultrasonography in distinguishing benign and malignant breast lesions. Therefore, SWE could be an additional tool to B-mode ultrasonography to identify malignant breast masses. For women who undergo repeated imaging, a combination of SWE and B-mode ultrasonography could enhance benign/malignant differentiation [30], [34]. SWE may be a viable and useful platform for detecting and characterizing focal lesions and for guiding the placement of interventional devices. As previously reported, ARFI imaging shows good performance in the identification of malignant liver lesions [7].

A strength of our study was the use of Fagan plot analysis to explore the clinical utility of SWE. At a pre-test probability for malignant breast masses of 25% (low clinical suspicion), the post-test probability of malignancy with a negative SSI result was 3%, which could be considered sufficient to rule out malignancy. At a pre-test probability for malignant breast masses of 50% (worst-case scenario), the post-test probabilities of malignancy with positive and negative SSI results were 83% and 10%, respectively, indicating the usefulness of this test. At a pre-test probability for malignancy of 75% (high clinical suspicion), the post-test probability for a malignant breast mass following a positive SSI result was 94%; thus, a positive SSI result could be considered sufficient for diagnosing malignancy. The ARFI technique was also very informative, with the probability of a correct diagnosis following a “positive” measurement reaching 91% for differentiating between benign and malignant breast masses when the pre-test probability was 50% and a probability of disease as low as 11% when a “negative” measurement was obtained, also indicating the usefulness of this test. At pre-test probabilities of malignancy of 25% (low clinical suspicion) and 75% (high clinical suspicion), ARFI could also be considered sufficient to rule out malignancy and sufficient for a diagnosis of malignancy, respectively.

These results, especially those of a “positive” SWE measurement, have been considered encouraging in individual studies. However, the studies included in our meta-analysis had high statistical heterogeneity. Heterogeneity in meta-analyses may be due to differences in test and/or study procedures, subject populations, study designs, or combinations of these factors. An additional source of heterogeneity in meta-analyses of diagnostic accuracy is the differences in the choice of a diagnostic threshold for a positive test result. Pooling “optimal” results from the studies included in our meta-analysis may have artificially increased the overall sensitivity and specificity. SWE might lead to the overdiagnosis of malignant breast masses, although this hypothesis needs to be confirmed. In clinical practice, however, malignant breast masses would be diagnosed relative to a single cut-off value. Therefore, we performed subgroup analyses according to SWE modes, but statistically significant heterogeneity still remained.

We also utilized meta-regression analysis to identify factors that may have caused the observed heterogeneity among the studies. Although covariates specific to patients and studies were examined, none were found to affect SSI and ARFI accuracy. In this context, a meta-analysis that included data from individual patients would allow the evaluation of the diagnostic performance of relevant cut-off values. Furthermore, the Deeks' funnel plot asymmetry test showed significant publication bias among these studies, suggesting that the findings presented here should be interpreted cautiously. Future large-scale studies are required to evaluate the efficacy of SWE in the classification of breast masses.

Our study had several limitations. First, unpublished studies were not identified, and no attempt was made to include articles in languages other than English. Second, although we identified 9 eligible studies, the analysis of the funnel plot suggested the possibility of publication bias. This may be due to our inclusion of only published English papers. In addition, there was significant heterogeneity among the included studies in the evaluation of SWE accuracy. Finally, the results were pooled for heterogeneous types of breast masses. In the SSI accuracy evaluation, 2 studies were conducted in Asia and 3 in Western countries; the studies evaluating ARFI performance were all conducted in Asia. There may be differences in the breast cancers of Western and Asian women. Because more than 4 studies are needed to conduct a meta-analysis using the MIDAS modules, subgroup analyses could not be performed in the evaluation of SSI for the identification of malignant breast masses.

In conclusion, our meta-analysis showed that SWE could be used to identify malignant breast masses, with a discriminant accuracy rate close to 90%, following a “positive” measurement. A “negative” measurement was also accurate and informative, with only 10% of patients having malignant disease. These results suggested the possibility that an increased proportion of women with benign masses can be reassured and discharged on the basis of the ultrasonography and SWE findings, without the need to undergo ultrasound-guided core biopsy. Future large-scale studies are required to confirm and extend these findings.

## Supporting Information

Figure S1 **Deeks' funnel plot asymmetry test for publication bias.** (A) Supersonic shear imaging for the differentiation between benign and malignant breast masses; (B) Acoustic radiation force impulse technique for the differentiation between benign and malignant breast masses. Sample size related to precision when there are unequal group sizes is more appropriately summarized by the effective sample size (ESS), where ESS = *(4n1n2)/(n1+n2)*. *n1*, numbers of non-diseased; *n2*, numbers of diseased.
(TIF)

Figure S2 **Analysis of the Fagan plot to evaluate the clinical utility of the acoustic radiation force impulse technique (ARFI) for differentiating benign from malignant breast masses.** (A) At a pre-test probability for malignant breast masses of 25% (low clinical suspicion), the post-test probability of malignancy with a negative ARFI result was 4%; this could be considered sufficient to rule out malignancy. (B) At a pre-test probability for malignant breast masses of 50% (worst-case scenario), the post-test probabilities of malignancy with positive and negative ARFI results were 91% and 11%, respectively, indicating the usefulness of this test. (C) At a pre-test probability for malignant breast masses of 75% (high clinical suspicion), the post-test probability of malignancy with a positive ARFI result was 97%; thus, a positive ARFI result could be considered sufficient for a diagnosis of malignancy. A Fagan plot consists of a vertical axis on the left showing the pre-test probability, an axis in the middle representing the likelihood ratio, and a vertical axis on the right showing the post-test probability. NLR, negative likelihood ratio; PLR, positive likelihood ratio.
(TIF)
